# Evaluation of the bacterial diversity among and within individual venous leg ulcers using bacterial tag-encoded FLX and Titanium amplicon pyrosequencing and metagenomic approaches

**DOI:** 10.1186/1471-2180-9-226

**Published:** 2009-10-27

**Authors:** Randall D Wolcott, Viktoria Gontcharova, Yan Sun, Scot E Dowd

**Affiliations:** 1 Southwest Regional Wound Care Clinic, Lubbock, TX 79410, USA; 2 Medical Biofilm Research Institute, Lubbock, TX 79407, USA; 3 Research and Testing Laboratory of the South Plains, Lubbock, TX 79407, USA

## Abstract

**Background:**

Approximately 1 out of every 100 individuals has some form of venous insufficiency, which can lead to chronic venous disease and Venous Leg Ulcer (VLU). There are known underlying pathologies which contribute to the chronic nature of VLU including biofilm phenotype infections.

**Results:**

Using pyrosequencing based approaches we evaluated VLU to characterize their microbial ecology. Results show that VLU infections are polymicrobial with no single bacterium colonizing the wounds. The most ubiquitous and predominant organisms include a previously uncharacterized bacteroidales, various anaerobes, *Staphylococcus*, *Corynebacterium*, and *Serratia*. Topological analysis of VLU show some notable differences in bacterial populations across the surface of the wounds highlighting the importance of sampling techniques during diagnostics. Metagenomics provide a preliminary indication that there may be protozoa, fungi and possibly an undescribed virus associated with these wounds.

**Conclusion:**

The polymicrobial nature of VLU and previous research on diabetic foot ulcers and surgical site infections suggest that the future of therapy for such wounds lies in the core of the logical and proven multiple concurrent strategy approach, which has been termed "biofilm-based wound care" and the use of individualized therapeutics rather than in a single treatment modality.

## Background

Approximately 600,000 Americans suffer from venous leg ulcers (VLU), which are extremely costly to manage and produce significant suffering [1]. Hippocrates believed that VLU were the bodies way to vent "evil humors" and advocated such ulcers should not be treated. His philosophy was that such ulcers should be allowed to express these evil humors naturally [2,3]. In spite of Hippocrates' beliefs, the modern clinical goal is to treat and cure VLU. Venous insufficiency is becoming epidemic with almost half of all females and one quarter of all males estimated to suffer from this disease [4]. It is generally agreed that chronic venous disease (CVD) is caused by persistent venous hypertension in the lower extremities stemming from a decay in the efficiency and performance of one-way valves in perforating, superficial or deep veins. Venous hypertension in the extremities, results in clinical changes leading from edema and pain (exacerbated upon standing for long periods of time) through lipodermatosclerosis, hyperpigmentation, hyperkeratosis and ultimately to a proclivity for the development of chronic VLU [1].

As the underlying pathology associated with CVD develops, ulcers typically start when the skin, in the area of fluid accumulation, becomes physically injured (e.g. cuts and abrasions). Because circulation is compromised due to associated pathologies, the effectiveness of the area to heal is reduced along with the overall functioning of the local immune system. The underlying pathological process, from the host perspective, still represents an area of developing hypotheses and has been reviewed recently in the literature [5]. A fully comprehensive, all encompassing understanding of the developmental mechanism related to why VLU remain chronic remains elusive and from a clinical perspective, Brem et al. stated "the exact mechanism underlying the formation of venous ulceration is unknown" [6].

VLU formation and their chronic nature is associated with a complex and multifactorial process. A primary factor contributing to the chronic nature of VLU is now known to be polymicrobial biofilm infection. The fact that many venous leg ulcers persist even after venous hypertension is adequately corrected clinically, is key evidence that this biofilm phenotype infection of the wound bed contributes significantly to the persistence associated with VLU. It is logical that this impaired host environment is extremely susceptible to opportunistic bacteria, which can then establish chronic infections. It also is logical that the contribution of biofilm to the production and persistence of VLU was overlooked until recently because its molecular footprint is so similar to the inflammation produced by or attributed solely to venous hypertension [7].

The current study was undertaken to better characterize the bacterial ecology of VLU using modern next-generation approaches [8-13]. Understanding the bacterial ecology of VLU associated biofilm is a critical next step in further evaluating the contribution of the wound microbiome to establishing and promoting the chronicity of VLU [14]. Using bTEFAP, metagenomic, quantitative PCR and the new bTEFAP Titanium based methods the bacterial diversity of 40 separate VLU, the overall metagenomic diversity in a pool of 10 VLU, and the topological bacterial diversity of 8 separate VLU are evaluated. This study represents one of the most comprehensive evaluations of microbial diversity in chronic wounds to date. The overall goal is to determine if VLU have the bacterial diversity between individual samples that we have shown with diabetic foot ulcers [9] and surgical site infections [13] and to do a preliminary screening of the total microbial diversity in these chronic wounds based upon a next-generation metagenomic approach. This metagenomic approach was also expected to help us to determine if there are any notable differences seen between a *de novo *approach to bacterial composition when compared to the 16s ribosomal DNA bTEFAP approach [15].

## Results and Discussion

### Diversity among 40 VLU

Using the bTEFAP methodology the diversity of 40 different VLU were individually evaluated. A total of 59,571 individual sequence reads longer than 200 bp were evaluated among the 40 samples with 46,993 sequences generating BLASTn hits against the bacterial database. The average sequence identity was 97.5%. A total of 16,029 sequences had identity below 97% suggesting they represented uncharacterized bacteria. The majority of these unknown organisms were most closely related based upon 16S sequence to *Bacterioides, Paludibacter, Pseudomonas*, *Finegoldia*, and *Corynebacterium *spp. These bacteria, which can be considered unknown or previously uncharacterized bacterial species, were identified based upon their closest identification and ranked at the genus, family or order level as appropriate. Only 101 of the total number of analyzed sequences fell below 80% identity and were not considered in subsequent analyses.

**Figure 1:**
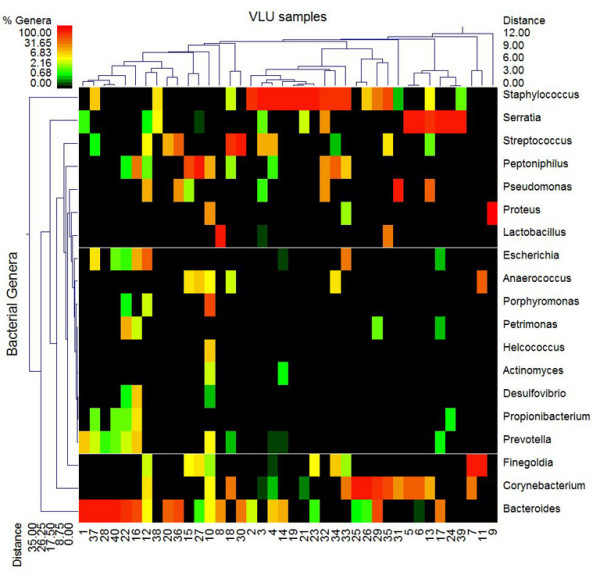
**Double dendogram of major genera in Venous Leg Ulcers**. This figure is a double dendogram describing the major genera detected among the 40 VLU samples. The heat map indicates the relative percentage of the given genera within each sample ID with a color legend and scale provided. The distance of the samples based upon weighted pair linkage and Manhattan distance methods with no scaling is provided at the top of the figure along with a distance score. The bacterial genera and the associated clustering are provided along the Y-axis and their associated distance scores indicated. The most determinative genera for clustering, based upon this analysis, are *Staphylococcus*, *Bacteroides*, *Serratia*, and *Corynebacterium *spp.

A total of 62 different genera (occurring in at least 2 of the wounds) were identified among the 40 wounds indicating a large relative diversity. The top 25 unique and most ubiquitous species (or closest taxonomic designation) are indicated in Table 1. The most ubiquitous genera were, in order and unknown *Bacteroides*, *Staphylococcus aureus*, and *Corynebacterium *spp The Bacteroides was only of marginal identity to any known *Bacteroides *species, thus represents a previously uncharacterized type of wound bacteria. Several genera were found in high percentage in individual wounds (Figure 1 dendogram). *Staphylococcus *spp. (which included primarily *S. aureus *but also several other coagulase negative species) predominated in 11 of the wounds, the unknown *Bacteroidetes *(which may represent a new genus based upon their identity) predominated in 8 of the wounds, *Serratia *(tenatively *marcescens*) was a predominant population in 6 of the wounds, *Streptococcus*, *Finegoldia*, *Corynebacterium *and *Peptoniphilus *spp. were the predominant genera in 2 wounds each, while *Proteus *and *Pseudomonas *spp. were the major population in one wound each. The remaining wounds were highly diverse with no overwhelmingly predominant populations. It is interesting that so many of these wounds were predominated by what are likely strict anaerobic bacteria with only very minor populations of facultative or strict aerobes. This suggests that such anaerobes might be contributing to the etiology of such biofilm infections. Figure 1 indicates there are a number of important functional equivalent pathogroups [9] associated with VLU. At a relative distance of 5 based upon the weighted-pair linkage and Manhattan distance we note there are 11 total clusters, which included 4 predominant clusters representing possible pathogroups [9]. It is also evident that *Staphylococcus*, *Serratia*, and *Bacterioides *are the defining variables for 3 of these 4 clusters. From this data we note that 53% of the populations were gram positive, 51.5% are facultative anaerobes, 30% were strict anaerobes, and 58% were rod shaped bacteria. Supplementary data (see additional file 1) provides a secondary comprehensive evaluation of the bacterial diversity in each of the 40 wounds.

**Table 1:** Evaluation of primary genera and species among the 40 VLU samples.

ID	Num of Samples	Avg %	St Dev	Min %	Max %
**Bacteroidales A**	22	28.2	34.8	0.1	98.1

***Staphylococcus aureus***	19	41.5	37.0	0.2	97.4

***Finegoldia magna***	14	12.3	26.8	<0.1	80.0

***Serratia marcescens***	12	43.0	42.6	0.1	99.0

***Staphylococcus aureus***	12	0.4	0.4	<0.1	1.1

***Corynebacterium *spp.**	11	22.7	26.8	0.1	90.2

***Peptoniphilus harei***	11	16.9	26.1	<0.1	82.0

***Escherichia coli***	8	6.9	9.4	0.1	26.0

***Anaerococcus prevotii***	8	4.1	7.4	0.1	22.2

***Pseudomonas aeruginosa***	7	19.4	30.7	0.1	86.7

***Staphylococcus *spp.**	7	2.0	4.5	0.1	12.1

***Propionibacterium acnes***	7	1.1	1.5	0.1	4.4

***Staphylococcus auricularis***	6	3.1	7.1	0.1	17.5

***Prevotella bryantii***	6	1.1	1.1	0.1	3.1

***Anaerococcus vaginalis***	5	2.7	3.2	0.2	6.7

***Corynebacterium *spp.**	4	10.5	11.7	0.2	26.1

***Staphylococcus haemolyticus***	4	8.2	8.6	0.4	16.7

**Bacteroidales B**	4	2.8	3.8	0.2	8.5

***Staphylococcus capitis***	4	0.4	0.4	0.1	1.0

***Streptococcus agalactiae***	3	48.2	42.2	0.2	79.6

***Porphyromonas somerae***	3	7.8	11.8	0.3	21.5

***Streptococcus agalactiae***	3	6.6	5.2	0.6	9.8

***Prevotella marshii***	3	1.7	2.5	0.1	4.5

***Streptococcus *spp.**	3	1.5	2.5	<0.1	4.3

***Actinomyces europaeus***	3	0.7	0.8	0.1	1.6

The primary identification based upon percent sequence identity as described in the materials and methods is indicated. For genera followed by spp. this indicates that resolution between multiple species of the same genera was not possible. The Bacteroidales designation represents the closest possible relationship for these previously uncharacterized bacteria. There is a second Bacteroidales (designated B), which also occurs in 4 of the wounds. Because these identifications are based upon average 250 bp such designations should be considered tentative at the species level. The results were however validated using quantitative PCR. The number of samples each bacteria was detected in is provided along with the average percent (avg %) among the positive samples, the standard deviation (st dev) and the range of percentages among the positive samples is provided.

As a confirmatory step for the bTEFAP diversity study we utilized a quantitative PCR wound diagnostic panel (Pathogenius diagnostics, Lubbock, TX), described previously [12,16]. A total of 8 of the VLU samples were chosen because they contained a predicted predominance of bacteria targeted by the qPCR wound panel. The results of the qPCR were provided to us in the form of relative ratios of each detected bacterium in the sample and these results compared to the corresponding bTEFAP bacterial ratio data. In short the percentages of the key bacteria detected using bTEFAP analysis were correlated (0.78, P = 0.001) with the relative percentages determined using qPCR. This provides an indication of the validity of the bTEFAP data.

### Metagenomics

We evaluated, using a bulk pyrosequencing metagenomics approach, a uniformly compiled pool of 10 VLU DNA extractions. A total of 178,610 individual reads were generated averaging 248 bp. There were 42,441 reads that could be assigned taxonomic designations. Of those reads assigned to a taxonomic designation the majority (30,141) fell into the chordata, which represents human genetic information confirmed based upon subsequence BLASTn and BLASTx designations to homo sapiens genomic data contained within NCBI. The remaining reads were utilized to generate an evaluation of the microbial population within these 10 VLU samples. There were 7,497 reads, which were assigned to bacteria, which was evaluated at the class level for the subsequent comparisons. Table 1 provides a comparative breakdown at the bacterial class level of bTEFAP analyses and the metagenomic analysis. There was good overall relationship (r-squared = 0.74) with what was predicted in the 10-sample VLU pool using metagenomic data and what was detected using the same 10 sample pool analyzed in our previous work using bTEFAP [15]. Interestingly, there was also a positive relationship at the same class taxonomic level between the 10-sample pool and the averages of the 40 VLU samples at the class level (Table 2).

**Table 2:** The 10 sample pool metagenomic analysis comparison to bTEFAP 10 sample pool and bTEFAP 40 sample averages at the taxonomic class level.

Class	bTEFAP 10 pool %	Metagenomics 10 pool %	bTEFAP 40 avg. %
**Bacilli**	4.5	4.6	29

**Gammaproteobacteria**	54	37.4	25

**Clostridia**	1.1	4.4	12

**Betaproteobacteria**	2.6	3.6	0.1

**Actinobacteria (class)**	1.1	19.1	12

**Alphaproteobacteria**	1.4	7.6	05

**deltaproteobacteria**	5.4	7.5	0.14

**Epsilonproteobacteria**	2	13	0.24

**Bacteroidetes**	10.5	6.1	17.9

**other**	17.2	8.6	3.5

This table shows the difference in metagenomic and 16s pyrosequencing approach described previously [15]. Also shown is the averages related to the 40 individual samples for comparison. The R-squared = 0.74 for correlation between bTEFAP and metagenomics at the class level in the 10 pooled samples.

Further analysis of the metagenomic data in relation to other microorganisms provided additional interesting information. A relatively high number of genes (2566) mapped to Apicomplexa (most closely related to *Plasmodium yoelii*) were detected. Fungi (most closely related to 3 yeast including *Candida albicans*, *Candida glabrata *and *Aspergillus *spp with some reads showing very distant relationships to *Yarrowia *spp and *Magnaporthe *spp) made up 668 reads. A total of 25 reads were designated as archaea. Another interesting finding within the metagenomic data was a high number of sequences (5450) most closely related to *Cyanobacteria*. This data could not be verified during subsequent analyses and was not noted in any of the bTEFAP datasets and evidence suggested it may be human mitochondrial sequence information (data not shown). However, the most surprising taxonomic relationship showed that 718 reads were most closely related to viruses, which was confirmed based upon homology to the "nr" and "nt" databases of NCBI. These included relationships to dsDNA viruses, no RNA stage primarily related to human herpes virus, human adenovirus, *Staphylococcus *phage, Gryllus bimaculatus virus, *Corynebacterium *phage, bacteriophage B3, and a high prevalence of Glypta fumiferanae ichnovirus related sequences. There were also a set of reads most closely related to retro-transcribing virus including tumor viruses, leukemia viruses, and Reticuloendotheliosis viruses. Represented within these designations were gene identifications related to gag-pol polyproteins, proteases, polymerases, envelope proteins, viral membrane proteins, capsid-associated proteins, carbohydrate binding proteins, fiber proteins, and immediate early genes. Because most of these reads were only distantly related to known virus, it is interesting to hypothesize about the presence of previously undiscovered virus associated with chronic wounds. It has been shown particularly in burn wounds that herpes virus I can cause infection and complications and even outbreaks within burn treatment units [17-19]. The presence of bacteriophage-related reads were to be expected considering the relatively high contribution of bacteria.

### Wound topology analysis

**Figure 2:**
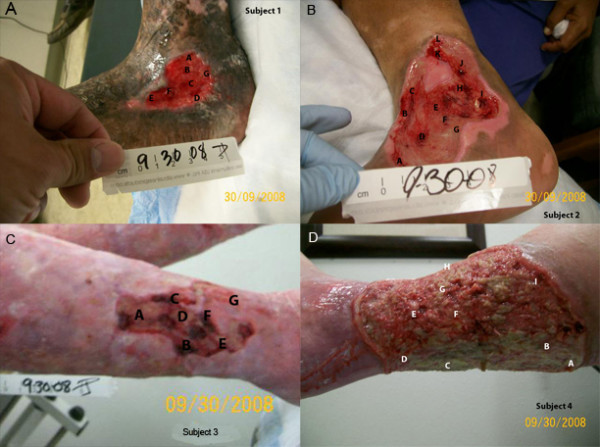
**Visual representation of venous leg ulcer sampling strategy**. Panels A-D. These figures provide examples of VLU with the transposed sampling locations for the topological bacterial diversity evaluation. The letters (e.g. A, B, C,...) indicate where each sample was gathered from each of these VLU. The detected bacterial diversity for each of these wounds is provided in Tables 3, 4, and 5.

We also evaluated a set of 4 VLU using both bTEFAP (Figure 2) and later a second set of 4 with the newest bTEFAP Titanium techniques. The goal of this analysis was to determine how homogeneous (or alternatively how heterogeneous) the bacterial ecology of wounds were across their surface. Our usual method, when we obtain samples for molecular diagnostics, indicates we debride larger areas that include center and edge regions and homogenize to obtain a global picture of the bacterial diversity. We continue to hold the assumption (backed up by most, if not all of the recent literature noted previously) that wounds are by definition very diverse in their microbial ecology among different samples, but within individual wounds the diversity is largely uniform. However, the question remained that (within a single wound) if we sample small discrete locations, rather than the typical larger areas we utilize clinically, would we see any variations in the populations? Figures 2 panels A, B, C, and D show the general sampling scheme for each of these samples with the corresponding bTEFAP data provided in Tables 3, 4, and 5 (data for subject 4 not included). The images and data associated with these 4 patients, which were evaluated using the original bTEFAP, provide a good indication of the topological diversity which may exist within individual wounds. Subject 1 had uniform occurrence of *Pseudomonas *(tentatively *aeruginosa*) across the entire wound with individual sites within the wound containing anaerobes including *Porphyromonas*, *Peptoniphilus*, *Finegoldia *and *Anaerococcus *spp. Subject 2 had relatively high divergence among each of the sampling sites. *Corynebacterium *was the most uniform bacteria along with *Pseudomonas *and *Proteus*. Several anaerobes were also very ubiquitous within the individual subsamples including *Anaerococcus*, *Clostridium *and *Peptoniphilus*. An unknown Enterobacteriacea was also observed in half of the subsamples. Subject 3 was interesting in that anaerobic *Peptoniphilus *was the most ubiquitous and predominant bacteria identified followed by *Corynebacterium*, *Peptostreptococcus*, *Pseudomonas*, *Staphylococcus*, and *Streptococcus*. This sample indicates the high divergence possible among such discrete subsamples. Subject 4 was the exception to the usual high bacterial diversity rule of chronic wounds and showed nearly 100 percent *Pseudomonas *in each of the sub samples. This topological evaluation of bacterial diversity indicates how important appropriate sampling is to fully characterize the global wound ecology.

**Table 3:** Results of topological bacterial diversity analysis for Subject 1 (Figure 2A).

Subject 1	A	B	C	D	E	F	G
	**Edge**	**Center**	**Center**	**Edge**	**Edge**	**Center**	**Edge**

***Pseudomonas***	**89.8**	**29.9**	**53.0**	**7.2**	**61.7**	**90.8**	**23.0**

***Serratia***	2.0	0.0	0.0	0.0	2.1	0.0	4.6

***Oxalobacteria***	2.0	**6.1**	0.0	0.0	4.3	0.0	0.0

***Porphyromonas***	0.0	**10.3**	**11.6**	**41.7**	0.0	0.0	**27.5**

***Peptostreptococcus***	0.0	0.0	0.0	**6.3**	0.0	0.0	1.1

***Peptoniphilus***	0.0	1.2	3.3	**10.4**	**8.5**	0.0	0.0

***Finegoldia***	0.0	1.2	1.9	**8.4**	0.0	0.0	1.6

***Fastidiosipila sp***	0.0	2.5	5.1	2.2	0.0	0.0	2.7

***Bordetella sp***	0.0	**31.0**	0.0	0.0	0.0	1.6	0.0

***Anaerococcus***	0.0	3.7	**9.3**	**5.0**	4.3	0.0	**10.2**

Percentages of each genera are indicated along with their location (A-G) based upon the map indicated in Figure 2A. The location designations (edge or center) are also provided.

**Table 4:** Results of topological bacterial diversity analysis for Subject 2 (Figure 2B).

Subject 2	A	B	C	D	E	F	G	H	I	J	K	L
**Location**	**E**	**E**	**E**	**C**	**C**	**C**	**E**	**C**	**C**	**C**	**E**	**E**

***Corynebacterium***	**87.5**	**19.0**	**20.1**	0.0	0.0	**16.9**	**27.7**	**81.4**	**11.4**	**53.3**	**71.9**	**93.9**

***Pseudomonas***	**5.3**	**15.0**	**27.0**	**71.5**	2.0	**7.2**	**7.8**	0.0	0.0	**20.0**	**6.0**	3.2

***Proteus***	1.8	**40.9**	**30.0**	0.0	0.0	**10.8**	**29.7**	0.0	**8.9**	**6.7**	4.3	0.0

***Enterobacteriaceae***	1.4	**18.1**	**5.7**	0.0	1.6	0.0	**12.4**	0.0	**5.7**	0.0	0.0	0.0

***Anaerococcus***	0.0	0.0	0.0	0.0	0.0	2.4	**7.9**	0.0	**6.5**	0.0	0.0	0.0

***Clostridia***	0.0	0.0	3.1	0.0	0.0	**20.5**	1.9	1.0	**8.9**	0.0	2.8	0.0

***Haemophilus***	0.0	0.0	0.0	0.0	4.5	0.0	0.0	0.0	0.0	0.0	0.0	0.0

***Peptoniphilus***	0.0	2.9	**6.3**	0.0	0.0	**38.6**	7.1	**11.5**	**50.4**	0.0	**9.1**	0.0

***Streptococcus***	0.0	0.0	0.0	0.0	**84.0**	0.0	0.0	0.0	0.0	0.0	0.0	0.0

***Serratia***	0.0	0.0	1.3	0.0	2.0	0.0	0.0	0.0	0.0	0.0	0.0	0.0

Percentages of each genera are indicated along with their location (A-L) based upon the map indicated in Figure 2B. The location designations (Edge or Center; E and C respectively) are also provided.

**Table 5:** Results of topological bacterial diversity analysis for Subject 3 (Figure 2C).

Subject 3	A	B	C	E	G	D	F
	**E**	**E**	**E**	**E**	**E**	**C**	**C**

*Peptoniphilus*	**32.1**	**62.5**	**49.4**	**54.2**	**13.9**	**44.0**	**9.6**

*Corynebacterium*	**10.7**	3.8	2.8	0.0	**15.6**	0.0	**13.1**

*Stenotrophomonas*	**14.2**	0.0	0.0	0.0	0.0	0.0	0.0

*Peptostreptococcus*	**7.1**	**6.2**	**7.7**	**6.1**	**6.5**	0.0	1.1

*Pseudomonas*	**17.8**	**7.5**	**17.1**	0.0	**21.3**	**12.0**	**11.8**

*Staphylococcus*	**7.1**	2.5	2.3	0.0	**31.0**	**20.0**	**41.0**

*Streptococcus*	3.6	3.8	1.5	0.0	3.8	4.0	1.7

*Acinetobacter*	0.0	0.0	2.3	0.0	3.3	0.0	4.4

*clostridia*	0.0	**7.5**	3.8	**5.5**	1.6	**8.0**	1.5

*Porphyromonas*	0.0	1.3	0.0	**23.7**	1.6	0.0	4.3

*Prevotella*	0.0	0.0	3.6	0.0	0.0	4.0	0.0

*Propionibacterium*	0.0	0.0	0.0	0.0	0.0	**8.0**	0.0

*Xanthomonas*	0.0	0.0	0.0	0.0	0.0	**12.0**	0.0

Percentages of each genera are indicated along with their location (A-G) based upon the map indicated in Figure 2C. The location designations (edge or center) are also provided.

Utilizing the new bTEFAP titanium technology a second topology evaluation was also conducted on 4 of the VLU patients. The new bTEFAP methods utilize the new Titanium chemistry for pyrosequencing, which increases the read length of individual sequences from an average of 250 bp to over 400 bp, utilize a single PCR step, and incorporate error reading polymerases. This new approach provides much better resolution at the individual species level and dramatically enhances our ability to characterize wound bacterial ecology. Four additional subjects were evaluated (See additional file 2). The results were similar to what we observed using the original bTEFAP method with the exception that we had more confidence in our ability to resolve certain populations at the species level. Subject 5 showed a high prevalence of *Pseudomonas aeruginosa *among the majority of the subsamples with notable populations of *Burkholdaria *spp (tentatively *cenocepacia*), an unknown Bacteroidales, and *Clostridium *spp (tentatively *hathewayi*). Subject 6 showed definite ubiquitous detection of *Pseudomonas aeruginosa *with notable populations of *Streptococcus parasanguinis *across the wound. Subject 7 showed a remarkable diversity and consistency across the entire wound with primary populations being *Staphylococcus aureus*, *Peptoniphilus harei*, *Staphylococcus capitis*, *Staphylococcus saprophyticus*, *Anaerococcus prevotii*, and *Finegoldia magna*. Finally Subject 8 also showed high consistency with major populations being *Streptococcus agalactiae*, *Corynebacterium striatum*, *Staphylococcus aureus*, with minor contributions in individual sites from *Pseudomonas aeruginosa *and *Corynebacterium simulans*. It should be noted that most of the wounds we have evaluated in the past have relatively high overall numbers of bacteria (>10^5 ^per mg debridement, based upon quantitative molecular methods) so even relatively low percentages of individual species such as 2% *Anaerococcus *spp. may potentially represents a large number of individual bacteria propagating within wound biofilms.

## Conclusion

Dowd et al [15] first used pyrosequencing to survey pooled samples of VLU, diabetic foot ulcers and decubitous ulcers and later did a more comprehensive survey of diabetic foot ulcers [9]. This study takes a similar but more comprehensive approach with VLU in order to better elucidate the individual ecologies in a large population of such chronic wounds. Here we show that individual wounds have distinct ecological footprints. We also show that within individual wounds there can be both significant site specific differences and relative uniformity in the bacterial ecology. The bottom line appears to be that each wound must be carefully evaluated and that no single pathogen is likely to be the causative agent of such infections. The wound care scientific and clinical opinion leaders have come to accepted the abundance of data showing that these polymicrobial biofilms represent a primary impediment to wound healing [9,14,20-30]. Based upon the current work and previous efforts we can deduce that the unique profiles of each individual wound indicate that a personalized approach to therapeutics combined with the multiple concurrent strategies of biofilm-based wound care [26] will revolutionize wound care. As Tom Pollard indicated in a commentary recently, biofilm-based wound care is " a significant shift in our whole approach to wound healing." [31]. Biofilm-based wound care combined with individualized therapeutic approaches and accurate rapid molecular diagnostics provides renewed found hope for those suffering with chronic wounds.

## Methods

### General sample collection methods

Patients were identified with VLU that have been persistent for over 6 months. These patients were enrolled in the study protocol after being educated and signing the informed consent protocol in compliance with Western Institutional Review Board approved protocols 56-RW-004 WIRB^® ^Protocol #20062347. Necessary details of the study including the protocols, guidelines and requirements were thoroughly explained to all the patients. Following these explanations, written consents was obtained in the presence of a third party witness. A copy of the consent form has been provided to journal editors. The patients were well informed that they have the right to opt out of the study at any time in spite of their written consent. VLU wound beds were debrided to remove superficial debris and cleansed with sterile saline. With aseptic precautions, sharp debridement was performed as part of standard care on the ulcer bed and subsequent samples collected into sterile 2 ml eppendorf tubes. The samples were immediately frozen at -80°C.

### Wound topology

Eight VLU chosen because they were particularly large and recalcitrant to healing had a MediRule II template (Briggs Corporation, Des Moines, IA) placed over the wound and the wound was outlined on the template grid. Multiple areas of the wound were chosen on the templates grid system and a variety of sample points chosen arbitrarily, which represented edge and center portions of the wound. Once these areas were marked on the template and the wound, the wound was then prepared. This was done by using normal saline irrigation along with a cotton gauze to gently remove surface debris. None of the wounds required local anesthesia and the areas that had been identified on the wound (as marked on the template) were then sampled. Individual sterile stainless steel curettes were used to debride an approximately 1.0 cm diameter sample of the biofilm down to the host tissue. Any bleeding at the sample sites was controlled with pressure. The patients reported no additional discomfort from the procedure. The samples were individually placed in separate sterile 2 cc Eppendorf tube (Fisher Scientific, Pittsburgh, PA), labeled with the patient's study accession number and grid location. The samples were then frozen at -80°C until subsequent molecular analysis.

### DNA extraction

After thawing, the debridement samples were centrifuged at 14,000 rpm for 30 seconds and resuspended in 500 μl RLT buffer (Qiagen, Valencia, CA) (with β-mercaptoethanol). A sterile 5 mm steel bead (Qiagen, Valencia, CA) and 500 μl sterile 0.1 mm glass beads (Scientific Industries, Inc., NY, USA) were added for complete bacterial lyses in a Qiagen TissueLyser (Qiagen, Valencia, CA), run at 30 Hz for 5 min. Samples were centrifuged briefly and 100 μl of 100% ethanol added to a 100 μl aliquot of the sample supernatant. This mixture was added to a DNA spin column, and DNA recovery protocols were followed as instructed in the QIAamp DNA Mini Kit (Qiagen, Valencia, CA) starting at step 5 of the Tissue Protocol. DNA was eluted from the column with 30 μl water and samples were diluted accordingly to a final concentration of 20 ng/μl. DNA samples were quantified using a Nanodrop spectrophotometer (Nyxor Biotech, Paris, France).

### Massively parallel bTEFAP and bTEFAP titanium

Bacterial tag-encoded FLX amplicon pyrosequencing (bTEFAP) was performed as described previously [9] at the Research and Testing Laboratory (Lubbock, TX.). The new bacterial tag-encoded FLX-Titanium amplicon pyrosequencing (bTETAP) approach is based upon similar principles to bTEFAP but utilizes Titanium reagents and titanium procedures and a one-step PCR, mixture of Hot Start and HotStar high fidelity taq polymerases, and amplicons originating from the 27F region numbered in relation to *E. coli *rRNA. The bTEFAP procedures were performed at the Research and Testing Laboratory (Lubbock, TX) based upon RTL protocols http://www.researchandtesting.com.

### Bacterial diversity data analysis

Following sequencing, all failed sequence reads, low quality sequence ends and tags were removed and sequences were depleted of any non-bacterial ribosome sequences and chimeras using custom software described previously [11] and the Black Box Chimera Check software B2C2 (described and freely available at http://www.researchandtesting.com/B2C2.html). Sequences less than 150 bp were removed for the original bTEFAP method and less than 350 bp for the bTEFAP titanium method. To determine the identity of bacteria in the remaining VLU sequences, sequences were first queried using a distributed BLASTn .NET algorithm [32] against a database of high quality 16s bacterial sequences derived from NCBI. Database sequences were characterized as high quality based upon the criteria of RDP ver 9 [33]. Using a .NET and C# analysis pipeline the resulting BLASTn outputs were compiled, validated using taxonomic distance methods, and data reduction analysis performed as described previously [9,11,13]. Rarefaction to estimate maximum diversity in wound using of 220 bp trimmed, non-ribosomal sequence depleted, chimera depleted, high quality reads was performed as described previously [8].

### Bacterial identification

Based upon the above BLASTn derived sequence identity (percent of total length query sequence which aligns with a given database sequence) and validated using taxonomic distance methods the bacteria were classified at the appropriate taxonomic levels based upon the following criteria. Sequences with identity scores, to known or well characterized 16S sequences, greater than 97% identity (<3% divergence) were resolved at the species level, between 95% and 97% at the genus level, between 90% and 95% at the family and between 80% and 90% at the order level. After resolving based upon these parameters, the percentage of each bacterial ID was individually analyzed for each wound providing relative abundance information within and among the VLU based upon relative numbers of reads within a given sample. Evaluations presented at a given taxonomic level, except species level, represent all sequences resolved to their primary genera identification or their closest relative (where indicated).

### Metagenomics

Metagenomic pyrosequencing reactions were performed at the Research and Testing Laboratory (Lubbock, TX). In short, DNA from a pool of 10 VLU preserved at -80°C, which had been previously analyzed using a 16s rDNA pyrosequencing microbial diversity approach [15] were further analyzed. DNA from these same 10 VLU samples were normalized and combined as described previously. Rather than perform bacterial 16s analysis as reported previously a metagenomic (or bulk sequencing) approach was performed using a half plate bulk sequencing reaction based upon FLX chemistry (Roche, Indianapolis, IN). In short, DNA was nebulized to generate 500 bp fragments, which were then ligated with Roche linkers and subsequently utilized to form a high-quality sequencing library, which was then subjected to massively parallel pyrosequencing (Roche, Indianapolis, IN). All methods were performed using manufacturers' suggested protocols. Following sequencing the individual sequence reads were screened to provide a final library of quality trimmed reads > 200 bp. These reads were then analyzed using IMG/M Expert Review metagenomics analysis system of the joint genome institute http://www.jgi.doe.gov. Individual reads were not assembled prior to analysis and only reads providing hits based upon IMG/M criteria [34,35] were utilized in the analyses. Due to HIPAA issues this data is not publically available but the microbial data has been deconvoluted and submitted to the SRA as indicated below.

### Quantitative PCR

Using a quantitative PCR wound diagnostic panel (Pathogenius diagnostics, Lubbock, TX), described previously [12,16] 8 of the 40 VLU samples, chosen because they contained a predicted predominance of bacteria targeted by the qPCR wound panel were evaluated. The results of the qPCR were provided in the form of relative ratios of each detected bacteria in the sample and these results compared to corresponding bTEFAP bacterial ratio data.

### Data submission and availability at NCBI

The data from the bTEFAP analyses and microbial metagenomic data are available in the National Center for Biotechnology Information' http://www.ncbi.nlm.nih.gov short read archive (SRA) under project accession number [GenBank:SRA008389.2/VLU].

### Basic Statistics

Statistics were performed using comparative functions and multivariate hierarchical clustering methods of NCSS 2007 (NCSS, Kaysville, Utah).

## Authors' contributions

SED co-conceived of the project, interpreted the data, and wrote the manuscript. YS performed laboratory procedures. VG assisted with data processing and analysis. RDW co-conceived of the project and helped write the manuscript. All authors have read and approved the final manuscript.

## Supplementary Material

Additional file 1 **Spreadsheet of bacterial genera detected among VLU**. The data file provides a complete compiled output of the bacterial genera detected among the VLU. Click here for file

Additional file 2 **Spreadsheet of VLU topology for subjects 5, 6, 7, and 8**. The data file provides a complete compiled output of the topology for the subjects 5, 6, 7, and 8 not included in the text. Click here for file
